# Remarks on struma ovarii in thyroidology: 3R; rare, risky, and redebated?

**DOI:** 10.1590/1806-9282.20250761

**Published:** 2026-07-10

**Authors:** Ilker Sengul, Demet Sengul

**Affiliations:** 1 Giresun University, Faculty of Medicine, Thyroidology Unit – Giresun, Turkey.; 2 Giresun University, Faculty of Medicine, Division of Endocrine Surgery – Giresun, Turkey.; 3 Giresun University, Faculty of Medicine, Department of General Surgery – Giresun, Turkey.; 4 Giresun University, Faculty of Medicine, Department of Pathology – Giresun, Turkey.

Dear Editor,

Struma ovarii (SO), a rare phenomenon of monodermal teratoma that emerges out of the first meiotic division of a single germ cell, per se, remains a challenging issue in Thyroidology, which requires a graceful approach^1,2,3,4,5,6,7,8,9,10^. We read with great interest the article by Erkan et al.^1^ entitled “*Struma Ovarii: Single Center Experience*.” This study presents a decade-long single-center experience of 19 cases diagnosed with SO, a monodermal teratoma predominantly composed of thyroid tissue, offering valuable insights into the clinical features, treatment, and follow-up of this rare ovarian tumor. The authors’ aim to contribute to the treatment algorithm by presenting a comprehensive case series of this rare entity, originating from the first meiotic division of a single germ cell and constituting approximately 2–3% of all ovarian tumors, is commendable. A teratoma is classified as SO when it contains more than 50% thyroid tissue^1^.

One of the significant strengths of this study is its inclusion of a relatively large case series of 19 patients with SO, a rare condition. Erkan et al.^1^ meticulously documented demographic data, presenting complaints, radiological findings, tumor sizes, laboratory data, surgical procedures, pathology reports, additional treatments, and follow-up information, which provides readers with a comprehensive overview of the clinical course of SO. The inclusion of malignant SO cases and the description of their management, involving unilateral salpingo-oophorectomy, total thyroidectomy, radioactive iodine (RAI) ablation, and L-thyroxine suppression, sheds light on the management of this uncommon occurrence. The authors’ suggestion that fertility-sparing surgery followed by postoperative adjuvant thyroidectomy and RAI ablation may be preferred for young women is a worthy consideration. The study found a higher rate of malignant SO compared to the reported range in the literature.

However, several limitations and potential areas for further exploration should be considered. The retrospective and single-center nature of the study may limit the generalizability of the findings. Of note, while such studies are valuable for rare conditions like SO, future multi-center and prospective studies could further contribute to the standardization of treatment approaches. As the authors noted, the preoperative diagnosis of SO is challenging due to the nonspecific nature of symptoms and the lack of a distinct radiological appearance. The fact that none of the patients in this study had a preoperative diagnosis of SO supports this observation. The absence of the characteristic “struma pearl” sonographic appearance in any of the cases is also noteworthy. Anecdotally, providing more detailed information regarding radiological findings, such as an analysis of how ultrasound and magnetic resonance imaging (MRI) features might differ between benign and malignant cases, could be beneficial for future research. As such, the management of malignant SO remains a topic of debate. The authors’ inference that risk assessment based on the histopathological features of the tumor might allow for fertility-sparing surgery to be sufficient is significant. However, this outcome warrants further investigation with more extensive case series and comparison groups. Furthermore, the study highlights that cancer antigen 125 (CA 125) levels were elevated in some benign cases. While elevated CA 125 is observed in some SO cases, it is not a specific marker. The association of SO with Pseudo-Meigs’ syndrome (ovarian mass, ascites, and pleural effusion) and elevated CA 125 has been reported in the literature. In this study, three patients had elevated CA 125 levels, with one being pregnant and two having moderate ascites. As the authors suggest, the increase in CA 125 might be attributed to the inflammatory process caused by ascites. Herein, further research is needed to clarify the role of CA 125 in SO diagnosis and its differentiation from other potential causes. Moreover, the increase in thyroglobulin (Tg) levels in the single malignant case that developed metastasis during follow-up and its subsequent normalization after metastasis surgery underscores the importance of Tg in monitoring malignant SO^1^.

In conclusion, this study gives a valuable contribution to the literature by presenting a single-center experience with SO. However, the retrospective nature and limited number of cases should be taken into account. Herewith, future multi-center and comparative studies can help establish more definitive guidelines for the diagnosis and optimal management, particularly of malignant SO. The authors’ suggestion regarding the potential sufficiency of fertility-sparing surgery with risk assessment in tumors lacking aggressive histopathological components is a noteworthy point for future consideration. This issue merits further investigation. We thank Erkan et al.^1^ for their valuable study on SO for thyroidologists.

## Data Availability

The datasets generated and/or analyzed during the current study are available from the corresponding author upon reasonable request.
